# Notch signaling sustains the expression of Mcl-1 and the activity of eIF4E to promote cell survival in CLL

**DOI:** 10.18632/oncotarget.4116

**Published:** 2015-05-12

**Authors:** Filomena De Falco, Rita Sabatini, Beatrice Del Papa, Franca Falzetti, Mauro Di Ianni, Paolo Sportoletti, Stefano Baldoni, Isabella Screpanti, Pierfrancesco Marconi, Emanuela Rosati

**Affiliations:** ^1^ Department of Experimental Medicine, Biosciences and Medical Embryology Section, University of Perugia, Perugia, Italy; ^2^ Department of Medicine, Hematology and Clinical Immunology Section, University of Perugia, Perugia, Italy; ^3^ Department of Life, Health and Environmental Sciences, Hematology Section, University of L'Aquila, L'Aquila, Italy; ^4^ Department of Molecular Medicine, Sapienza University of Rome, Rome, Italy

**Keywords:** chronic lymphocytic leukemia, Notch, Mcl-1, eIF4E, cell survival

## Abstract

In chronic lymphocytic leukemia (CLL), Notch1 and Notch2 signaling is constitutively activated and contributes to apoptosis resistance. We show that genetic inhibition of either Notch1 or Notch2, through small-interfering RNA, increases apoptosis of CLL cells and is associated with decreased levels of the anti-apoptotic protein Mcl-1. Thus, Notch signaling promotes CLL cell survival at least in part by sustaining Mcl-1 expression. In CLL cells, an enhanced Notch activation also contributes to the increase in Mcl-1 expression and cell survival induced by IL-4.

Mcl-1 downregulation by Notch targeting is not due to reduced transcription or degradation by caspases, but in part, to increased degradation by the proteasome. Mcl-1 downregulation by Notch targeting is also accompanied by reduced phosphorylation of eukaryotic translation initiation factor 4E (eIF4E), suggesting that this protein is another target of Notch signaling in CLL cells.

Overall, we show that Notch signaling sustains CLL cell survival by promoting Mcl-1 expression and eIF4E activity, and given the oncogenic role of these factors, we underscore the therapeutic potential of Notch inhibition in CLL.

## INTRODUCTION

Notch signaling is involved in various cellular processes, including cell fate specification, differentiation, proliferation, and apoptosis. Abnormal Notch signaling is oncogenic in several cancers, including hematologic malignancies [1]. Disregulated Notch signaling has also been associated with chronic lymphocytic leukemia (CLL), a frequent adult leukemia characterized by the accumulation of CD19^+^/CD5^+^ B lymphocytes resistant to apoptosis [2]. The involvement of Notch in CLL has been recently demonstrated by findings that a *NOTCH1* PEST domain mutation, generating a highly active truncated protein, and affecting up to 10-15% of patients, is associated with poor prognosis, disease progression and refractoriness to chemotherapy [3-7]. Previous evidence that constitutive activation of Notch1 and Notch2 signaling contributes to apoptosis resistance in CLL also underscores the importance of Notch in this leukemia [8, 9], encouraging further investigation of its therapeutic potential. Indeed, a better understanding of the mechanisms involved in the anti-apoptotic signaling of Notch in CLL cells may provide insight for designing future Notch-targeted therapies.

In cancer, Notch signaling prevents apoptosis through different networks, involving cell cycle and survival pathways, and interactions with mitochondria. Notch suppresses p53 [10] or JNK function [11] as well as the expression of the pro-apoptotic proteins Bax, Bim and Noxa [12, 13]. Notch increases the activation of the pro-survival PI3K/AKT [14] and NF-kB pathways [15] and the expression of the anti-apoptotic proteins Bcl-2 and Bcl-xL [12], stabilizes the apoptosis inhibitor protein XIAP [16] and induces mitochondrial integrity and functions [17]. Some of these functions are mediated by the transcriptional activity of Notch-intracellular domain (ICD) [10, 14, 15], which, after Notch-ligand interactions, is released from the membrane to the nucleus. Other functions involve a non-canonical Notch-ICD-activated signaling which operates in the cytoplasm [11, 16] and can also converge on the mitochondria by promoting cell survival [17].

A crucial role in controlling mitochondrial integrity and apoptosis is played by the balance between pro-apoptotic and anti-apoptotic Bcl-2 family members [18]. In CLL as well as in other hematologic malignancies, the over-expression of the anti-apoptotic Mcl-1 and Bcl-2 proteins is one of the major causes of apoptosis resistance [19, 20], poor prognosis [21, 22] and chemoresistance [23-25]. Mcl-1 and Bcl-2 bind and sequester the pro-apoptotic proteins Bax and Bak blocking their ability to form pores in the mitochondrial membrane with the consequent release of cytochrome c into the cytoplasm. Degradation of Mcl-1 frees Bax and Bak allowing their polymerization and activating apoptosis [18]. Mcl-1 is a short-lived protein tightly regulated by transcriptional [26, 27], translational [28, 29], and degradation mechanisms [30, 31]. Interestingly, Mcl-1 mRNA translation is highly dependent on the eukaryotic initiation factor 4E (eIF4E), a key component of the mRNA cap-binding complex, which preferentially enhances translation of a subset of mRNAs with complex 5^'^ untranslated regions, such as those of Mcl-1 and several other transformation-related and survival proteins [32-34]. eIF4E has been associated with cancer development and progression, and proposed as an important therapeutic target [35, 36]. Recently, it has been demonstrated that CLL cells also express high levels of eIF4E, and that its pharmacologic targeting increases *in vitro* fludarabine cytotoxicity, suggesting an involvement of eIF4E in chemoresistance of these cells [37]. Considering the critical role of Mcl-1 and eIF4E in CLL and in other malignancies, in this study, we investigated whether Mcl-1 and eIF4E are targets of the anti-apoptotic Notch signaling in CLL.

## RESULTS

### Notch1 and Notch2 downregulation decreases viability of CLL cells from different patient subgroups

**Figure 1:**
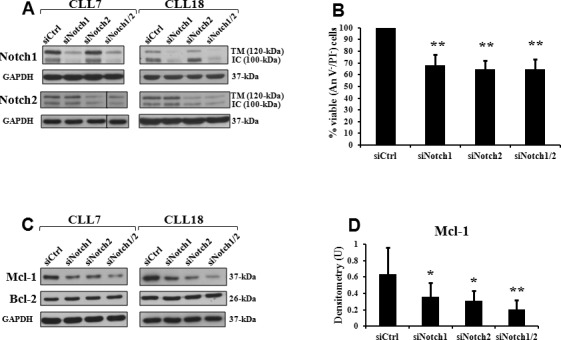
Notch1 and Notch2 silencing decreases cell viability and expression of Mcl-1 protein in CLL cells CLL cells were transfected with control siRNA (siCtrl), Notch1 siRNA (siNotch1), Notch2 siRNA (siNotch2) or combined siNotch1 and siNotch2 (siNotch1/2) as described in “siRNA transfection” and then cultured in complete medium for 72 hours. **A.**, **C.** Western blot analysis of Notch1, Notch2, Mcl-1 and Bcl-2 expression was performed on 15 μg whole-cell lysates separated on a 7.5% SDS-PAGE. The antibodies used for Notch1 and Notch2 recognized the 120-kDa transmembrane/cytoplasmic subunit (TM) and the 100-kDa active intracellular domain (IC). Protein loading was assessed by reprobing the blots with an anti-GAPDH antibody. Vertical line inserted in Notch2 blot of CLL7 indicates a repositioned gel lane. Data of CLL7 and 18 are representative of six samples. **D.** The blots of Mcl-1 were subjected to densitometric analysis and densitometry units (U) were calculated relative to GAPDH. Data are the mean ± SD of six samples. *P < 0.05, **P < 0.01 (each siNotch transfection condition *versus* siCtrl) according to Student *t* test. **B.** Cell viability was evaluated by flow cytometric analysis of Annexin V/PI (An V/PI) staining. Viability (An V^−^/PI^−^) of siCtrl cells was set to 100%. Data are the mean ± SD of six samples. **P < 0.01 (each siNotch transfection condition *versus* siCtrl) according to Student *t* test.

We used small-interfering RNA (siRNA) and nucleofection to silence expression of Notch1 and Notch2 in all 22 patients included in the study. Table 1 gives clinical and biological characteristics of CLL patients. Downregulation of the expression of each Notch receptor, achieved at different levels in all samples examined (Table 2), did not affect the levels of the other receptor (Figure 1A), suggesting that the expression of each of them is independent of the other. As previously reported [8, 9] and shown in Table 2 and Figure 1B, silencing of either Notch1 (siNotch1) or Notch2 (siNotch2) decreased, to a similar extent, CLL cell viability compared with cells transfected with control siRNA (siCtrl). This effect was observed in 18 of 22 samples (Table 2), suggesting that both receptors contribute to CLL cell survival in the vast majority of patients (81.8%). The four patients with Notch-independent viability (CLL4, 17, 20, 22) did not belong to any specific subgroup regarding clinical and biological characteristics (Table 1). Decrease in cell viability induced by silencing of each receptor varied among the different CLL samples, ranging from 17.7 to 65.2% for Notch1 and from 16.1 to 51.4% for Notch2 (Table 2). However, similar responses were observed irrespective of Binet stage, previous therapy, *IgVH* mutational status and ZAP70 and CD38 expression (Table 3), suggesting that Notch targeting is effective in CLL cells despite the presence of adverse prognostic factors. Even in the three samples with *NOTCH1* PEST domain mutation (CLL1, 7, 11; Table 1), either Notch1 or Notch2 downregulation reduced CLL cell viability at levels similar to those observed in *NOTCH1*-unmutated samples (Table 3). These results suggest that *NOTCH1* mutation does not influence the sensitivity of CLL cells to Notch targeting, at least when it is harboured by a small fraction of leukemic cells, as indicated by the low *NOTCH1* mutant allele burden detected in all three mutated samples examined (Table 1).

**Table 1:** Characteristics of CLL patients

Patients	Binet stage	Previous treatment^a^	*IgVH* status^b^	ZAP70 expression^c^	CD38 expression^d^	*NOTCH1* status(% mutant allele burden)^e^	Cytogenetic alterations^f^
**CLL1**	B	no	Unm	+	−	Mut (1.9)	Normal
**CLL2**	C	yes	Unm	+	−	Unm	del 11q22-23 del 13q14
**CLL3**	A	no	Unm	+	ND	Unm	ND
**CLL4**	C	yes	Unm	+	+	Unm	del 11q22-23 del 13q14
**CLL5**	C	yes	Unm	+	+	Unm	del 13q14 del 14q32
**CLL6**	A	no	Mut	+	−	Unm	Normal
**CLL7**	B	yes	Unm	+	+	Mut (2.4)	del 11q22-23
**CLL8**	B	yes	Unm	+	+	Unm	ND
**CLL9**	C	yes	Unm	−	−	Unm	ND
**CLL10**	C	yes	Mut	−	−	Unm	del 13q14 del 17p13
**CLL11**	B	yes	Unm	−	−	Mut (1.3)	del 11q22-23 del 13q14 del 14q32
**CLL12**	C	yes	Unm	+	−	Unm	del 11q22-23 del 13q14
**CLL13**	A	yes	Unm	+	+	Unm	Normal
**CLL14**	C	yes	Unm	+	−	Unm	del 14q32
**CLL15**	B	no	Mut	−	−	Unm	Normal
**CLL16**	C	yes	Mut	+	+	Unm	ND
**CLL17**	A	no	Mut	−	−	Unm	del 13q14
**CLL18**	A	no	Mut	−	−	Unm	del 13q14
**CLL19**	C	yes	Mut	+	−	Unm	ND
**CLL20**	B	yes	Mut	−	+	Unm	Normal
**CLL21**	B	yes	Unm	+	−	Unm	del 13q14 del 14q32
**CLL22**	B	no	Mut	+	−	Unm	Normal

Mut, mutated; Unm, unmutated; ND, not determined.

a Treated patients had not received treatment for at least 3 months before the study.

b Mutated was defined as having a frequency of mutations >2% from germline *VH*.

c Positivity refers to detection of >20% ZAP70+/CD19+.

d Positivity refers to detection of >20% CD38+/CD19+.

e c.7544_7545delCT in *NOTCH1* exon 34.

f Assessed by FISH.

**Table 2:** Effect of Notch1 and Notch2 silencing on CLL cell viability and Mcl-1 protein expression

Patients	siRNA	Receptor	% reduction in Notch expression		% reduction in CLL cell viability	% reduction in Mcl-1 protein expression
			TM	IC		
CLL 1						
	siNotch1	Notch1	55.0	67.0	32.6	39.0
	siNotch2	Notch2	51.2	61.1	17.9	38.0
CLL 2						
	siNotch1	Notch1	76.0	53.8	19.8	none
	siNotch2	Notch2	30.2	38.1	16.2	none
CLL 3						
	siNotch1	Notch1	98.0	99.3	65.2	70.0
	siNotch2	Notch2	71.0	64.0	51.4	29.8
CLL 4						
	siNotch1	Notch1	87.0	69.5	none	none
	siNotch2	Notch2	84.0	64.4	none	none
CLL 5						
	siNotch1	Notch1	57.0	80.1	32.7	36.9
	siNotch2	Notch2	43.7	65.3	35.1	51.0
CLL 6						
	siNotch1	Notch1	61.5	52.8	38.1	44.4
	siNotch2	Notch2	84	78	43.7	50.9
CLL 7						
	siNotch1	Notch1	73.2	89.1	29.7	44.2
	siNotch2	Notch2	69.0	56.7	23.1	32.6
CLL 8						
	siNotch1	Notch1	78.2	72.1	29.3	36.0
	siNotch2	Notch2	52.0	47.3	24.8	23.2
CLL 9						
	siNotch1	Notch1	67.0	73.0	33.8	27.7
	siNotch2	Notch2	81.0	72.5	39.5	34.0
CLL 10						
	siNotch1	Notch1	78.0	58.0	25.0	none
	siNotch2	Notch2	75.0	51.0	28.4	none
CLL 11						
	siNotch1	Notch1	48.0	59.0	20.8	22.9
	siNotch2	Notch2	41.0	54.0	38.2	41.7
CLL 12						
	siNotch1	Notch1	51.0	65.0	31.5	none
	siNotch2	Notch2	35.3	40.1	17.1	none
CLL 13						
	siNotch1	Notch1	31.0	35.2	17.7	23.0
	siNotch2	Notch2	75.7	61.2	16.1	48.0
CLL 14						
	siNotch1	Notch1	79.0	72.0	26.1	92.0
	siNotch2	Notch2	88.0	98.3	20.7	80.0
CLL 15						
	siNotch1	Notch1	44.4	52.0	58.4	41.9
	siNotch2	Notch2	72.7	59.8	34.9	26.0
CLL 16						
	siNotch1	Notch1	58.0	69.0	43.9	59.4
	siNotch2	Notch2	65.0	71.0	36.1	50.0
CLL 17						
	siNotch1	Notch1	47.1	53.0	none	none
	siNotch2	Notch2	69.0	71.0	none	none
CLL 18						
	siNotch1	Notch1	89.0	74.5	23.9	52.9
	siNotch2	Notch2	73.9	52.8	31.3	71.6
CLL 19						
	siNotch1	Notch1	36.0	31.3	18.4	32.0
	siNotch2	Notch2	56.4	45.1	20.5	35.4
CLL 20						
	siNotch1	Notch1	95.3	71.0	none	none
	siNotch2	Notch2	50.7	45.4	none	none
CLL 21						
	siNotch1	Notch1	68.3	75.3	33.6	21.0
	siNotch2	Notch2	57.3	61.3	44.3	44.0
CLL 22						
	siNotch1	Notch1	45.6	61.0	none	none
	siNotch2	Notch2	81.9	82.3	none	none

Expression of Notch1 and Notch2 and Mcl-1 was analyzed by western blot in CLL cells transfected with Notch1 (siNotch1), Notch2 (siNotch2) or control siRNA (siCtrl). The band intensities of Notch1 and Notch2 (TM and IC) and Mcl-1 were quantified by densitometric analysis and normalized to GAPDH. Values represent the percentage reduction of Notch1 and Mcl-1 expression in siNotch1 cells, and of Notch2 and Mcl-1 expression in siNotch2 cells, compared with siCtrl cells. Viable CLL cells were quantified by flow cytometry using Annexin V/PI staining, and the percentage reduction in viability of siNotch1 and siNotch2 cells was calculated relative to siCtrl. None indicates reductions in cell viability or Mcl-1 expression lower than 5%.

**Table 3:** Notch1 and Notch2 silencing decreases CLL cell viability independently of clinical characteristics and prognostic factors

Binet stages and prognostic factors	Number of patients	% Viability related to siCtrl (mean ± SD)^a^			
		siNotch1	*P*	siNotch2	*P*
*Binet stage*					
A	5	70.5 ± 23.7	0.778 (A vs B)	71.3 ± 20.4	0.594 (A vs B)
B	8	73.8 ± 18.2	0.974 (B vs C)	76.9 ± 16.1	0.924 (B vs C)
C	9	74.1 ± 11.8	0.707 (C vs A)	76.2 ± 12.2	0.580 (C vs A)
*Previous treatment^b^*					
No	7	68.1 ± 24.6		74.2 ± 20.0	
Yes	15	75.5 ± 11.4	0.337	75.9 ± 13.1	0.814
*IgVH status^c^*					
Unm	13	71.2 ± 14.1		73.5 ± 14.2	
Mut	9	76.1 ± 20.0	0.505	78.0 ± 16.9	0.501
*ZAP70 expression^d^*					
Negative	7	76.1 ± 19.2		75.0 ± 16.6	
Positive	15	71.8 ± 15.6	0.579	75.4 ± 15.0	0.954
*CD38 expressione^e^*					
Negative	14	73.8 ± 14.2		74.7 ± 14.3	
Positive	7	77.4 ± 15.8	0.599	80.4 ± 14.4	0.402
*NOTCH1 status^f^*					
Unm	19	73.3 ± 17.7		75.6 ± 15.9	
Mut	3	72.3 ± 6.2	0.925	73.6 ± 10.5	0.839

Mut, mutated; Unm, unmutated.

a Percentage of viable cells determined by Annexin V/PI staining in siNotch1 or siNotch2 transfected CLL cells related to siCtrl cells as 100% viability.

b Treated patients had not received treatment for at least 3 months before the study.

c Mutated was defined as having a frequency of mutations >2% from germline *VH*.

d Positivity refers to detection of >20% ZAP70+/CD19+.

e Positivity refers to detection of >20% CD38+/CD19+.

f c.7544_7545delCT in *NOTCH1* exon 34.

The evidence that Notch1 and Notch2 exert redundant effects in promoting CLL cell survival prompted us to examine the effect of combined Notch1 and Notch2 silencing. We simultaneously transfected CLL cells with Notch1 and Notch2 siRNA (siNotch1/2), and performed these experiments in six CLL samples (patients 6, 7, 9, 11, 16, 18), selected to include patients with different clinical and biological characteristics. Results showed that although the combined siNotch1/2 transfection efficiently downregulated the expression of both receptors (Figure 1A), it did not further decrease CLL cell viability with respect to transfection of each single receptor (Figure 1B).

### Notch1 and Notch2 silencing decreases Mcl-1 but not Bcl-2 protein expression in CLL cells

Based on the evidence that Mcl-1 and Bcl-2 proteins are highly expressed in CLL cells and play a crucial role in apoptosis resistance and CLL pathogenesis [19, 23], we analyzed the effect of Notch downregulation on the expression of these proteins (*n* = 22). We found that in 15 of the 18 samples where Notch1 and Notch2 downregulation reduced CLL cell viability, there was a decrease in Mcl-1 levels compared with siCtrl cells, whereas in the four samples with Notch-independent viability, Mcl-1 levels remained unchanged (Table 2, Figures 1C and 1D). When we transfected CLL cells with combined siNotch1/2 (*n* = 6), we observed that reduction of Mcl-1 levels was more pronounced (Figures 1C and 1D). In contrast, in all 22 patients, Bcl-2 levels were not affected by either each single or combined Notch receptor downregulation (Figure 1C and data not shown). Altogether, these data indicate that apoptosis of CLL cells induced by Notch silencing involves downregulation of Mcl-1 but not Bcl-2 expression.

### Combined Notch1 and Notch2 silencing prevents the increase in Mcl-1 levels and cell viability induced in CLL cells by IL-4

**Figure 2:**
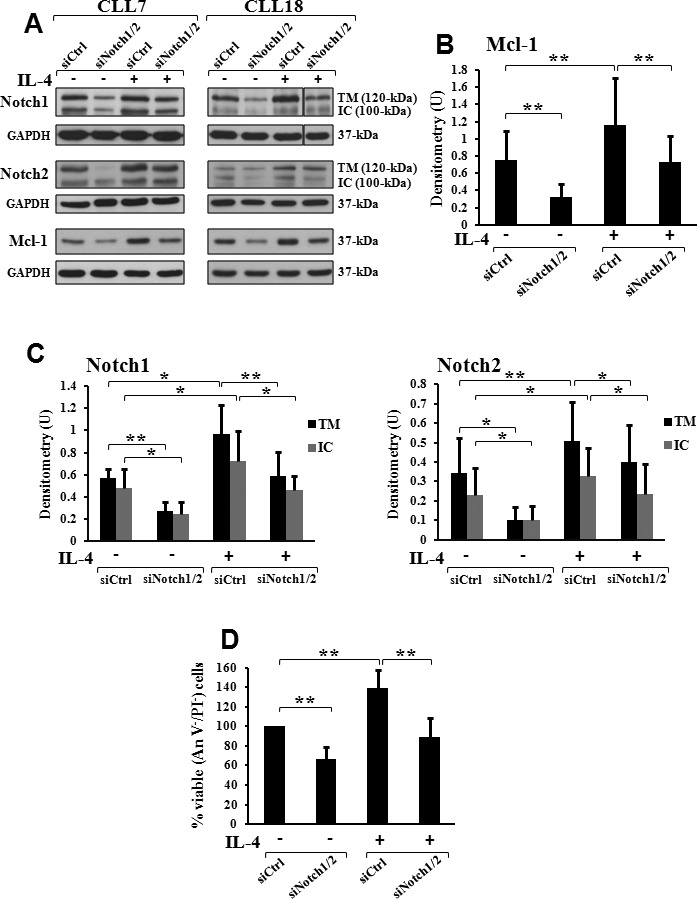
Combined Notch1/2 silencing prevents the increase in Mcl-1 levels and cell viability induced by IL-4 in CLL cells CLL cells, transfected with control siRNA (siCtrl) or combined Notch1 and Notch2 siRNA (siNotch1/2) as described in “siRNA transfection”, were cultured for 72 hours in complete medium with or without 25 ng/ml IL-4 (*n* = 6). **A.** Expression of Notch1, Notch2 and Mcl-1 was analyzed as described in Figure 1A,C. Vertical line inserted in Notch1 blot of CLL18 indicates a repositioned gel lane. The blots of Mcl-1 **B.** and those of Notch1 and Notch2 **C.** were subjected to densitometric analysis, and densitometry units (U) were calculated relative to GAPDH. **A.** Data of CLL7 and 18 are representative of six samples. **B.**, **C.** Data are the mean ± SD of six samples. *P <0.05, **P < 0.01 calculated by Student *t* test. **D.** Cell viability was evaluated by flow cytometric analysis of Annexin V/PI (An V/PI) staining. Viability (An V^−^/PI^−^) of IL-4-untreated siCtrl cells was set to 100%. Data are the mean ± SD of six samples. **P < 0.01 calculated by Student *t* test.

Several micro-environmental stimuli have been shown to promote *ex vivo* CLL cell survival by increasing Mcl-1 expression [38, 39]. The involvement of Notch signaling in Mcl-1-mediated CLL cell survival induced by the microenvironment has never been explored. We examined whether the T-cell derived cytokine IL-4, known inducer of cell survival and Mcl-1 expression in CLL [39], enhanced Notch expression in promoting these effects, and if so, whether this increase was required for Mcl-1-mediated CLL cell survival induced by IL-4. CLL cells, transfected with siCtrl or combined siNotch1/2, were cultured for 72 hours with or without IL-4, and then examined for Notch1, Notch2 and Mcl-1 expression, and cell viability/apoptosis (*n* = 6). In agreement with previous studies [39], we found that in siCtrl cells, IL-4 increased both Mcl-1 levels (Figures 2A and 2B) and cell viability (Figure 2D). In siCtrl cells, IL-4 significantly upregulated also Notch1 and Notch2 expression (Figures 2A and 2C), and interestingly, combined Notch1/2 silencing partially abrogated the increase in both Mcl-1 expression and cell viability induced by the cytokine (Figures 2A-2D). These results indicate that in CLL cells, IL-4 enhances Notch expression and that this event is required to induce the increase in Mcl-1-mediated cell survival.

### Mcl-1 downregulation by Notch silencing is not due to transcriptional control or degradation by caspases but in part to degradation by proteasome

**Figure 3:**
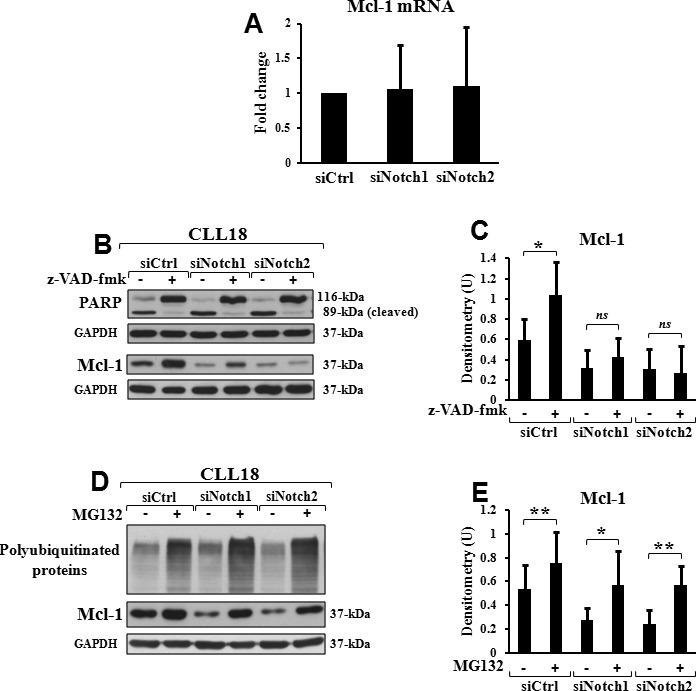
Mcl-1 downregulation by Notch silencing partially depends on proteasome degradation **A.** Mcl-1 downregulation by Notch silencing is independent of reduced transcription. CLL cells were transfected with control siRNA (siCtrl), Notch1 siRNA (siNotch1) or Notch2 siRNA (siNotch2) as described in “siRNA transfection” and then cultured in complete medium for 72 hours (*n* = 6). Mcl-1 mRNA levels were evaluated by real-time PCR, normalized to GAPDH and represented as fold change with respect to siCtrl cells. Data are the mean ± SD of six samples. Differences between each siNotch transfection and siCtrl were not significant. **B.**, **C.** Mcl-1 downregulation by Notch silencing is independent of degradation by caspases. siCtrl, siNotch1 or siNotch2 transfected cells were cultured for 72 hours in complete medium with 50 μM pan-caspase inhibitor z-VAD-fmk or 0.005% DMSO as control (*n* = 6). **B.** PARP cleavage, indicator of caspase activity, and Mcl-1 expression were analyzed by western blot on 15 μg whole-cell lysates. Protein loading was assessed by reprobing the blots with an anti-GAPDH antibody. Data of CLL18 are representative of six samples. **C.** The blots of Mcl-1 were subjected to densitometric analysis, and densitometry units (U) were calculated relative to GAPDH. Data are the mean ± SD of six samples. ^*^P < 0.05; *ns*, not significant (z-VAD-fmk-treated cells *versus* DMSO-treated cells in each transfection condition) according to Student *t* test. **D.**, **E.** Mcl-1 downregulation by Notch silencing partially depends on degradation by proteasome. siCtrl, siNotch1 or siNotch2 transfected cells were cultured for 72 hours in complete medium additioned, during the last 4 hours, with 2.5 μM proteasome inhibitor MG132 or 0.001% DMSO as control (*n* = 6). **D.** Accumulation of polyubiquitinated proteins, indicator of proteasome inhibition, and Mcl-1 expression were analyzed by western blot on 15 μg whole-cell lysates. Protein loading was assessed by reprobing the blots with an anti-GAPDH antibody. Data of CLL18 are representative of six samples. **E.** The blots of Mcl-1 were subjected to densitometric analysis, and densitometry units (U) were calculated relative to GAPDH. Data are the mean ± SD of six samples. *P < 0.05, **P < 0.01 (MG132-treated cells *versus* DMSO-treated cells in each transfection condition) according to Student *t* test.

Mcl-1 protein expression is regulated at multiple levels, including transcription, translation and degradation [26-31]. To define whether the reduced Mcl-1 expression induced by Notch targeting was due to transcriptional inhibition, we analyzed Mcl-1 mRNA expression by real-time PCR (*n* = 6). In siNotch1 as well as in siNotch2 cells, Mcl-1 mRNA levels were similar to those observed in siCtrl cells (Figure 3A), suggesting that Notch silencing reduces Mcl-1 expression at posttranscriptional level.

It has been shown that Mcl-1 can be degraded by caspases during apoptosis [40]. To test whether Notch silencing decreased Mcl-1 levels through degradation by caspases, we cultured siNotch1, siNotch2 and siCtrl cells with or without the pan-caspase inhibitor z-VAD-fmk (*n* = 6). Results showed that whereas in siCtrl cells, z-VAD-fmk increased Mcl-1 levels, in siNotch1 as well as in siNotch2 cells treated with z-VAD-fmk, Mcl-1 levels continued to be downregulated, although PARP cleavage, an indicator of caspase activation, continued to be inhibited as in siCtrl cells (Figures 3B and 3C). These results suggest that reduction in Mcl-1 expression induced by Notch targeting was independent of caspase activation.

Another mechanism controlling Mcl-1 protein expression is proteasomal degradation [26, 30, 31]. To determine whether the decrease in Mcl-1 levels induced by Notch targeting was due to proteasomal degradation, we tested the effect of the proteasome inhibitor MG132 (*n* = 6). It was added to siNotch1, siNotch2 and siCtrl cells during the last 4 hours of the 72-hour post transfection culture, and its action was demonstrated by the accumulation of polyubiquitinated proteins, an indicator of proteasome inhibition (Figure 3D). In siCtrl cells, MG132 increased Mcl-1 levels, indicating that in CLL cells cultured *ex vivo*, Mcl-1 is degraded by proteasome. In both siNotch1 and siNotch2 cells, MG132 prevented the loss of Mcl-1 protein which returned to levels observed in siCtrl cells (Figures 3D and 3E). These results suggest that Mcl-1 downregulation by Notch targeting depends, at least in part, on increased degradation by proteasome.

### Mcl-1 downregulation by Notch silencing is accompanied by reduced eIF4E phosphorylation

**Figure 4:**
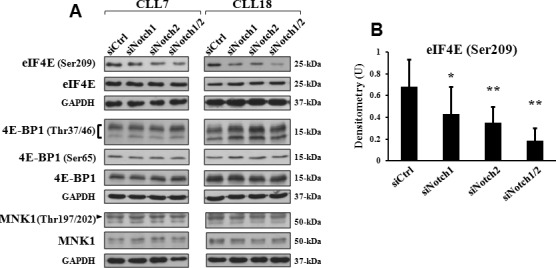
Mcl-1 downregulation by Notch silencing is associated with reduced phosphorylation of eIF4E CLL cells were transfected with control siRNA (siCtrl), Notch1 siRNA (siNotch1), Notch2 siRNA (siNotch2) or combined siNotch1 and siNotch2 (siNotch1/2), as described in “siRNA transfection” and then cultured in complete medium for 72 hours (*n* = 6). **A.** Expression and phosphorylation of eIF4E (Ser209), 4E-BP1 (Thr37/46 and Ser65) and MNK1 (Thr197/202) were analyzed by western blot on 15 μg whole-cell lysates, using antibodies able to detect total and phosphorylated forms. Protein loading was assessed by reprobing the blots with an anti-GAPDH antibody. Data of CLL7 and 18 are representative of six samples. **B.** The blots of phosphorylated eIF4E (Ser209) were subjected to densitometric analysis, and densitometry units (U) were calculated relative to total eIF4E. Data are the mean ± SD of six samples. *P < 0.05, **P < 0.01 (each siNotch transfection condition *versus* siCtrl) according to Student *t* test.

Mcl-1 mRNA translation is highly dependent on the activity of the eukaryotic initiation factor 4E (eIF4E) [33], a key translation regulator associated with tumorigenesis [35, 36]. eIF4E is indeed frequently over-expressed and over-activated in human cancers, and acts at a converging point of relevant oncogenic pathways. One pathway regulating eIF4E activity is mediated by the MAPK-interacting kinases 1 (MNK1) and MNK2, which are targets of the Ras/Raf/MAPK signaling and directly phosphorylate eIF4E at Ser209, a crucial event for its oncogenic activity [41]. Another pathway is mediated by the eIF4E-binding protein 1 (4E-BP1), a target of the PI3K/AKT/mTOR signaling, which, when is hypophosphorylated, prevents eIF4E activity through inhibitory interactions [42]. Based on these observations, we examined whether in CLL cells, Mcl-1 downregulation by Notch silencing was accompanied by effects on the expression and phosphorylation of eIF4E (Ser209), MNK1 (Thr197/202), and 4E-BP1 (Thr37/46 and Ser65). Consistent with previous studies [37], our results showed that all examined CLL cells (*n* = 6) expressed high levels of both total and phosphorylated eIF4E forms. eIF4E phosphorylation, but not expression, was reduced by either Notch1 or Notch2 targeting, and to a greater extent, by combined Notch1/2 targeting (Figures 4A and 4B). In contrast, expression and phosphorylation levels of MNK1 and 4E-BP1 were not affected by either each single or combined Notch receptor downregulation (Figure 4A). These results indicate that Notch signaling controls eIF4E activity, but in doing this, it does not involve MNK1 and 4E-BP1 regulation. These results, along with recent evidence that eIF4E contributes to CLL cell survival [37], also suggest that eIF4E activity is another target of the anti-apoptotic Notch signaling in CLL, in addition to Mcl-1.

## DISCUSSION

In the present study, we have identified in the anti-apoptotic Mcl-1 protein and in the key translational regulator eIF4E, two targets of the pro-survival activity of Notch signaling in CLL cells. These findings are interesting because Mcl-1 and eIF4E are oncogenic in several malignancies [26, 35], including CLL. In this leukemia, both proteins are important mediators of cell survival [19, 20, 37], with Mcl-1 which is closely associated with adverse prognosis [21, 22] and chemoresistance [23, 24].

Specifically, we have shown that the increase in CLL cell apoptosis induced by either Notch1 or Notch2 genetic inhibition is accompanied by Mcl-1 protein downregulation. In contrast, the levels of Bcl-2 protein remained unaffected indicating that Bcl-2 is not a target of the anti-apoptotic signaling of Notch in CLL cells and that Mcl-1 reduction is not due to a general effect of Notch silencing. This observation along with the evidence that the decrease in Mcl-1 levels induced by Notch silencing was observed in the vast majority of CLL cells with Notch-dependent viability (83.3%), but not in CLL cells whose viability was independent of Notch, suggest that Mcl-1 is important for Notch-mediated CLL cell survival. Thus, one of the mechanisms by which Notch1 and Notch2 sustain CLL cell survival is by maintaining the constitutive high levels of Mcl-1.

In CLL cells, several pro-survival signals have been implicated in promoting Mcl-1 expression, including those mediated by STAT3 [27], NF-kB [43], and Syk/AKT pathways [20, 44]. This is the first evidence that in CLL cells, Mcl-1 expression is also sustained by Notch signaling. This effect of Notch is not due to transcriptional regulation because reduction in Mcl-1 levels induced by Notch1 and Notch2 targeting is not accompanied by changes in Mcl-1 mRNA expression. Mcl-1 downregulation by Notch targeting is also independent of cleavage by caspases, but, as shown in studies of pharmacologic proteasome inhibition, it is partially due to increased Mcl-1 proteasome-mediated degradation, suggesting that Notch signaling controls this process, and contributes to stabilize Mcl-1 levels by interfering with it. Whether in CLL cells, Notch receptors act directly on Mcl-1 protein to stabilize it, as Notch1 does to stabilize XIAP protein [16], or indirectly, by influencing some of the pathways which regulate Mcl-1 proteasome-mediated degradation in these cells, including the Syk/PKCδ [45] and AKT/GSK3 [46] pathways, remains to be defined.

Furthermore, the evidence that Mcl-1 downregulation by Notch targeting is accompanied by a decreased activity of eIF4E, an essential factor for Mcl-1 translation, also suggests that Notch signaling may control Mcl-1 expression by regulating its biosynthesis. Further studies will be needed to define this point and to clarify the mechanisms whereby Notch signaling sustains eIF4E activity in CLL cells, given that Notch targeting does not have any effect on the phosphorylation levels of 4E-BP1 and MNK1, which in other cell types, are two key upstream regulators of eIF4E activity [41, 42].

The impact of Notch signaling on Mcl-1 expression in CLL cells is also supported by the evidence that the combined Notch1/2 downregulation induces a higher reduction of Mcl-1 levels than that induced by downregulating each single receptor. However, these results seem discordant with the observation that combined Notch1/2 silencing does not enhance CLL cell apoptosis induced by downregulating each single receptor. A possible explanation is that the anti-leukemic activity of a strong Notch downregulation is limited by compensatory survival mechanisms, suggesting that inhibition of Notch signaling alone is not sufficient to kill all leukemic cells. This is consistent with the evidence that in several malignancies, the best clinical activity of Notch-targeted therapies was observed when the specific Notch inhibitors, including γ-secretase inhibitors (GSI) or monoclonal antibodies to Notch receptors or Notch ligands, were administered in combination with conventional chemotherapy or other targeted agents [47]. In line with this evidence, even in CLL cells, it has been recently demonstrated that the clinically relevant GSI PF-03084014 improves the pro-apoptotic effect of fludarabine [48]. This occurs because Notch inhibition overcomes the resistance mechanisms activated in CLL cells by fludarabine [23], including increased NF-kB activation [48] and Mcl-1 expression [21]. In this context, a critical role of Notch in regulating NF-kB pathway has been previously described in T-cell leukemia [15, 49] and a role of Notch in controlling Mcl-1 expression is here demonstrated in CLL.

Another important aspect of this study is that Notch signaling also contributes to Mcl-1 accumulation induced by survival micro-environmental stimuli. Specifically, we demonstrate that IL-4, known to induce in CLL cells an increased Mcl-1-mediated cell survival [39], also enhances Notch1 and Notch2 activation. Interestingly, combined Notch1/2 downregulation partially prevents the increase in both CLL cell survival and Mcl-1 expression, suggesting that Notch targeting, in addition to reducing the constitutive Mcl-1 levels, is also able to prevent Mcl-1 accumulation induced by micro-environmental stimuli. This effect of Notch downregulation is important because in CLL, the major resistance mechanisms to current chemotherapy, including Mcl-1 expression, are highly favored by the microenvironment [38].

Overall, Notch signaling sustains CLL cell survival by promoting Mcl-1 expression and eIF4E activity. These findings along with the evidence that both Mcl-1 and eIF4E contribute to survival and chemotherapy resistance of CLL cells highlight the importance to target Notch signaling for CLL treatment, especially in combination with agents whose poor efficacy is mainly due to the elevated Mcl-1 expression and eIF4E activity detected in these leukemic cells.

## MATERIALS AND METHODS

### Patients

Twenty-two CLL patients entered this study. Diagnoses of CLL were based on Stanford criteria defined by the National Cancer Institute-sponsored Working Group [50], and clinical staging was based on the Binet classification [51]. This study was approved by the local Ethics Committee, and all patients signed informed consent in accordance with the Declaration of Helsinki.

### CLL cell isolation

Peripheral blood mononuclear cells were isolated from heparinized blood of CLL patients by Ficoll density-gradient centrifugation (Nycomed, Oslo, Norway). Monocytes were removed by plastic adherence, and T cells by sheep erythrocyte rosetting. All CLL samples contained more than 96% CD19^+^/CD5^+^ CLL cells, as assessed by flow cytometry (EPICS-XL-MCL; Beckman Coulter, Fullerton, CA).

### CLL clinical laboratory characteristics

*IgV_H_* mutations, CD38 surface and ZAP70 intracellular expression were analyzed as previously reported [8]. Cytogenetic abnormalities were examined by fluorescent in situ hybridization (FISH) using probes for chromosomes 11, 12, 13, 14 and 17. *NOTCH1* exon 34 mutations were analyzed as previously reported and the percentage of mutant allele burden was determined using a semi-quantitative assay on the basis of Genescan analysis [4]. Table 1 gives clinical and biological characteristics of CLL patients.

### siRNA transfection

CLL cells (12 × 10^6^) were resuspended in 100 μl Cell Line Solution Kit V (Lonza Group Ltd, Basel, Switzerland) with ON-TARGETplus SMARTpool small interfering RNA (siRNA) to Notch1 (0.5 μM), Notch2 (0.5 μM) or ON-TARGETplus siCONTROL nontargeting pool as negative control (all from Dharmacon RNA Technologies, Lafayette, CO). In the case of combined Notch1 and Notch2 silencing, 0.25 μM per siRNA was added. Cells were then transfected with the Amaxa Nucleofector II device (program U-013) and cultured for 72 hours in 12-well plates in complete medium consisting of RPMI 1640 supplemented with 10% heat-inactivated fetal bovine serum (Hyclone Laboratories, Logan, UT), 2 mM L-glutamine, 100 U/ml penicillin and 100 μg/ml streptomycin (all from Invitrogen, Milan, Italy). In some experiments, transfected cells were incubated with 25 ng/ml recombinant human IL-4 (Immunotools, Friesoyte, Germany), 50 μM pan-caspase inhibitor z-VAD-fmk or 2.5 μM proteasome inhibitor MG132 (both from Calbiochem, La Jolla, CA). The inhibitors z-VAD-fmk and MG132 were dissolved in DMSO and diluted in complete medium at the used concentrations. DMSO concentrations, which did not exceed 0.005%, did not affect CLL cell responses. IL-4 and z-VAD-fmk were added at the beginning of the 72-hour post transfection culture and maintained until cell collection, whereas MG132 was added during the last 4-hour culture.

### Analysis of cell viability and apoptosis

Cell viability and apoptosis were assessed by flow cytometric analysis (EPICS-XL-MCL) after Annexin V-fluorescein isothiocyanate/propidium iodide staining, performed using a commercial kit (Immunotech, Beckman Coulter) according to the manufacturer's instructions.

### Western blot analysis

Whole-cell lysates were extracted in RIPA buffer. Equal amounts of proteins were separated by 7.5% SDS-PAGE and transferred to nitrocellulose membranes, which, after blocking, were incubated with primary antibodies to: Notch1 (clone bTAN20) and Notch2 (clone C651.6DbHN), developed by Spyros Artavanis-Tsakonas, obtained from DSHB developed under the auspices of the NICHD, and maintained by Iowa University; Mcl-1 (Santa Cruz Biotechnology, Santa Cruz, CA); Bcl-2 (DakoCytomation, Milan, Italy); phospho-eIF4E (Ser209), total eIF4E, phospho-4E-BP1 (Ser65), phospho-4E-BP1 (Thr37/46), total 4E-BP1, phospho-MNK1 (Thr197/202), total MNK1 and PARP (Cell Signaling Technology, Beverly, MA); ubiquitinated proteins (BIOMOL Research Laboratories, Plymouth Meeting, PA) and GAPDH (Sigma-Aldrich, St. Louis, MO). Signals were detected using appropriate horseradish peroxidase-conjugated secondary antibodies and the ECL system (GE Healthcare, Milan, Italy). Densitometric analysis was performed using Quantity One software (Bio-Rad, Milan, Italy).

### Real-time quantitative PCR

Total RNA was isolated using Trizol (Invitrogen) and 1 μg reverse-transcribed using RT-kit plus (Nanogen Advanced Diagnostics, Milan, Italy). Real-time quantitative PCR was performed with PCR Master Mix Power SYBR Green (Applied Biosystems, Warrington, UK), using the 7900HT Fast Real-Time PCR System (Applied Biosystems). The primer sequences used for *MCL-1* were forward: 5′-GAG ACC TTA CGA CGG GTT-3′ and reverse: 3′-TTT GAT GTC CAG TTT CCG-3′ (Invitrogen). Relative fold change was normalized to *GAPDH* and calculated using the 2^−ΔΔCt^ method.

### Statistical analysis

Statistical differences between mean values were evaluated using the Student *t* test. The minimal level of significance was *P <* 0.05.
